# Ultrasonographic features of feline renal neoplasia: a retrospective study on 187 cases

**DOI:** 10.1177/1098612X261432299

**Published:** 2026-03-03

**Authors:** Alessia Cordella, Jennifer Lenz, Mileva Bertal, Manabu Kurihara, Agustina Anson, Federica Serafini, Helen Dirrig, Linda Dillenbeck, Stefano De Arcangeli, Wilfried Mai

**Affiliations:** 1 Department of Clinical Sciences and Advanced Medicine, University of Pennsylvania School of Veterinary Medicine, Philadelphia, PA, USA; 2 Department of Diagnostic Imaging, École Nationale Vétérinaire d’Alfort, Maisons-Alfort, France; 3 Department of Molecular Biomedical Sciences, College of Veterinary Medicine, North Carolina State University, Raleigh, NC, USA; 4 Department of Clinical Sciences, Cummings School of Veterinary Medicine at Tufts University, North Grafton, MA, USA; 5 Department of Veterinary Clinical Sciences, College of Veterinary Medicine, Purdue University, West Lafayette, IN, USA; 6 Department of Clinical Science and Services, The Royal Veterinary College, London, UK; 7 Department of Environmental and Radiologic Health Sciences, Colorado State University Veterinary Teaching Hospital, Fort Collins, CO, USA

**Keywords:** Kidney, ultrasonography, lymphoma, carcinoma, oncology, azotemia

## Abstract

**Objectives:**

Ultrasonography plays a crucial role in diagnosing feline renal diseases, including neoplasia. The aims of this study were to describe ultrasonographic features of renal neoplasia in cats and to investigate potential differences between tumor types.

**Methods:**

In this multicenter retrospective study, ultrasonographic images of feline kidneys with cytologically/histologically confirmed renal neoplasia were reviewed. For each kidney, ultrasonographic characteristics (renal length, presence and appearance of a mass, nodules, hypoechoic subcapsular rim, pelvic distension, retroperitoneal effusion) were recorded and compared for each tumor type.

**Results:**

A total of 187 cats (373 kidneys) were included. Tumor types were lymphoma (n = 118, 63%), carcinoma (n = 53, 28.5%), sarcoma (n = 10, 5%), adenoma (n = 3, 2%), histiocytic sarcoma (n = 2, 1%) and nephroblastoma (n = 1, 0.5%). Bilateral disease (*P* <0.001) and other organ involvement (*P* = 0.026) were more frequent in lymphoma. A single mass was more frequent in carcinoma (*P* <0.001). Masses were more frequently hypoechoic in lymphoma (81%) and sarcoma (86%) than in carcinoma (40%) (*P* = 0.001). In kidneys with masses, a hypoechoic subcapsular rim was more frequent in lymphoma (*P* = 0.004). In kidneys without mass lesions, kidneys with lymphoma (mean size 51.3 ± 9.8 mm) were significantly larger (*P* = 0.02) than those with carcinoma (mean size 46.1 ± 6.4 mm) and sarcoma (mean size 42.8 ± 8.9 mm).

**Conclusions and relevance:**

Lymphoma was the most common renal neoplasia, followed by carcinoma. Some ultrasonographic features, including bilateral involvement, single masses, multiple nodules, hypoechoic subcapsular rim and severity of nephromegaly, can help differentiate feline renal tumor types.

## Introduction

Primary renal tumors are rare in cats, accounting for less than 2% of all feline neoplasms, with lymphoma being the most frequently reported, followed by carcinoma.^1–3^ Other tumor types, including sarcomas, nephroblastoma, adenoma, histiocytic sarcoma and leiomyoma, are rarely reported in the feline literature.^4–11^ Distinguishing between these tumor types is of fundamental importance because they exhibit significant differences in their biological behavior and therefore require distinct treatment strategies. The recommended treatment for renal lymphoma in cats is multiagent chemotherapy, with commonly used regimens being COP-based protocols (vincristine, cyclophosphamide and prednisolone).^12–16^ In contrast, nephrectomy is the preferred treatment option for cats with renal carcinoma, with possible prolonged survival times reported in those that survive to discharge.^17^

Abdominal ultrasound (US) is the most used imaging modality for evaluating the feline urinary tract, as it offers a non-invasive, fast and effective tool to assess renal size, shape, contour, internal architecture and retroperitoneal space.^18–20^ Cytopathology (and/or histopathology) is necessary for confirmation of the diagnosis, but US can provide crucial information regarding the extent of the disease and concurrent abnormalities, and can be used for monitoring disease progression and response to treatment. Certain US features, such as diffuse renal enlargement, parenchymal nodules and hypoechoic subcapsular rim, can raise suspicion for renal neoplasia in cats, irrespective of the tumor type.^17–23^ Few studies describe the US findings of feline renal lymphoma^21,24^ and feline renal carcinoma,^17,23^ with isolated case reports mentioning the imaging appearance of more uncommon tumor types.^8,9,11^ However, comprehensive studies detailing the ultrasonographic features of various renal tumor types in a large feline population are currently lacking.

Therefore, the primary objective of this study was to describe the ultrasonographic findings in cats with confirmed renal neoplasia and to determine whether these characteristics vary among different tumor types. A secondary objective was to evaluate potential differences in clinicopathological changes among cats presenting with different renal neoplasia.

## Materials and methods

For this multicenter retrospective study, the electronic medical records of cats examined at the university teaching hospitals of seven different institutions (University of Pennsylvania, École Nationale Vétérinaire d’Alfort, North Carolina State University, Cummings School of Veterinary Medicine at Tufts University, Purdue University, Royal Veterinary College and Colorado State University) between 2010 and 2024 were reviewed. Cats were included if a cytological or histological confirmation of renal tumor was present (including cytology from fine-needle aspiration and/or histology from Tru-cut biopsy, whole organ after nephrectomy or necropsy) and if US images of the urinary tract (still images and/or recorded cineloops) were available for review.

Exclusion criteria included a non-definitive diagnosis or uncertain tumor type, US performed after the treatment protocol was initiated, and absent, incomplete or poor-quality US images.

Data collected included signalment (breed, age and sex) and, when available, serum creatinine and blood urea nitrogen (BUN) concentrations and urine specific gravity (USG). Serum creatinine and BUN concentrations were considered high when above the reference interval provided by the laboratory; USG was considered low when it was below 1.035.^25^ Based on medical records (combination of cytological/histological results, imaging, surgical reports and/or follow-up), the renal involvement in the neoplastic process for each cat was classified as unilateral or bilateral.

When additional imaging was present, including thoracic radiographs or CT, abdominal CT or head/neck CT, concurrent involvement of other organs was also recorded, along with cytological/histological confirmation, when available.

### Ultrasound image review

All US images of the kidneys were reviewed by a European College of Veterinary Diagnostic Imaging board-certified radiologist (AC). Recorded US findings for each kidney included nephromegaly (defined as maximum renal length >44 mm^18^), presence of a hypoechoic subcapsular rim^21^ and whether there was a solitary mass or multiple nodules. In the case of a single mass, its localization (cranial, mid- or caudal parenchyma, within/around the pelvis, subcapsular or occupying the whole kidney), echogenicity in relation to the renal cortex (hypo/iso/hyperechoic or mixed) and echotexture (homogeneous/heterogeneous) were recorded. Furthermore, pelvic distension (with dimensions in millimeters measured in the transverse plane) and perinephric effusion and/or steatitis were also recorded for each kidney. Pelvic distension was considered mild when it measured less than 4 mm in the transverse plane,^26^ moderate when it measured greater than 4 mm with no distension of the pelvic recesses, and severe when it was greater than 4 mm and pelvic recesses were also dilated. The renal length, measured in either a sagittal or dorsal plane, and the maximum dimensions of the focal lesions were also recorded.

### Statistical analysis

Statistical analyses were performed to evaluate the relationship between renal US findings and tumor type. Ultrasonographic variables were primarily analyzed at the kidney level, as imaging features such as mass presence, echogenicity, subcapsular rim and pelvic dilation are organ-specific and may differ between kidneys within the same patient. Analyses evaluating bilateral renal involvement and associations with clinicopathological variables were performed at the patient level, with each cat contributing a single observation. Categorical variables were compared among the groups (when five or more subjects were present) using the χ^2^ test or Fisher’s exact test. Continuous data were analyzed using the Kruskal–Wallis test, with Dunn’s post hoc pairwise comparisons to identify specific group differences. To determine which US features independently predicted tumor diagnosis between lymphoma and carcinoma, a binomial logistic regression was performed. Pearson’s χ^2^ tests were used for two-group comparisons (lymphoma vs carcinoma), to examine the association between serum creatinine and BUN concentrations, USG and tumor type. A logistic regression analysis was performed within the lymphoma subgroup to evaluate the effect of patient age, bilateral renal involvement and lymphoma US presentation (diffuse or focal) on renal impairment. Lymphoma lesions were categorized into diffuse (characterized by generalized nephromegaly and a subcapsular rim) or focal/multifocal (manifested by the presence of a mass or nodules).

Analyses were performed using Stata 18.0 (StataCorp), and statistical significance was set as *P* <0.05.

## Results

Of the 216 cats initially identified, 187 met the inclusion criteria and were included in the study. As a result, 373 kidneys (one of the included cats had only one kidney, having been a previous renal donor) were reviewed. The median age at diagnosis was 11 years (range 1–21); 100/187 (53%) cats were males (93 castrated) and 87/187 (47%) were females (82 spayed). Most of the included cats were domestic shorthairs (n = 154); other included breeds were domestic longhair (n = 12), Siamese (n = 5), Maine Coon (n = 4), Bengal (n = 2) and one each of Abyssinian, Turkish Angora, British Shorthair, Havana Brown, Ocicat, Persian, Ragdoll, Devon Rex, Sphynx and Tonkinese.

### Diagnosis

Renal lymphoma was the most common diagnosis (118/187, 63%), followed by carcinoma (53/187, 28.5%). Of the 187 cats, 10 (5%) cats had a renal sarcoma, three (2%) were diagnosed with adenoma, two (1%) with histiocytic sarcoma and one (0.5%) with nephroblastoma.

Lymphoma was diagnosed by cytology in 107/118 (91%) cats, carcinoma in 31/53 (58%) and sarcoma in 7/10 (70%). The remaining 11 lymphomas, 22 carcinomas and three sarcomas were diagnosed by histology. The two included cases of histiocytic sarcoma were diagnosed by cytology, while all three cases of adenoma and the single nephroblastoma were diagnosed by histology.

No significant differences were found in breed, sex or age among the tumor groups.

### Bilateral vs unilateral renal involvement

The renal changes were bilateral in 100 cases and unilateral in 87 (including the cat with a single kidney). Bilateral renal involvement was observed significantly more frequently in cats with lymphoma (*P* <0.001): both kidneys were involved in 89/118 (75%) cats with lymphoma, 6/53 (11%) cats with carcinoma, 4/10 (40%) cats with sarcoma and 1/2 (50%) cat with histiocytic sarcoma. Adenomas and nephroblastoma were unilateral in all cases.

### US findings

Of the 373 kidneys evaluated, 287 were diagnosed with neoplasia and included in the statistical analysis (the remaining 86 kidneys were the contralateral ones in cats with unilateral renal tumors).

The frequency of renal US findings in the different tumor types is summarized in Table 1.

**Table 1:** Ultrasonographic characteristics of the kidneys included in each tumor group (the total number corresponds to the number of kidneys)*

	Lymphoma (n = 207)	Carcinoma (n = 59)	Sarcoma (n = 14)	Histiocytic sarcoma (n = 3)	Adenoma (n = 3)	Nephroblastoma (n = 1)
Nephromegaly	162	36	7	2	1	1
Single mass	42	42	7	0	3	1
Multiple nodules	59	2	6	2	0	0
Hypoechoic subcapsular rim	155	26	5	1	0	0
Pelvic distension	95	28	5	3	0	1
Retroperitoneal steatitis/effusion	144	36	10	2	0	1

* Lymphomas are more frequently presented with nephromegaly and hypoechoic subcapsular rim, carcinomas (and adenomas and nephroblastoma) more often with a single mass, sarcomas with either a single mass or multiple nodules, and histiocytic sarcomas with multiple nodules

#### US features when a single renal mass was present

**Figure 1:**
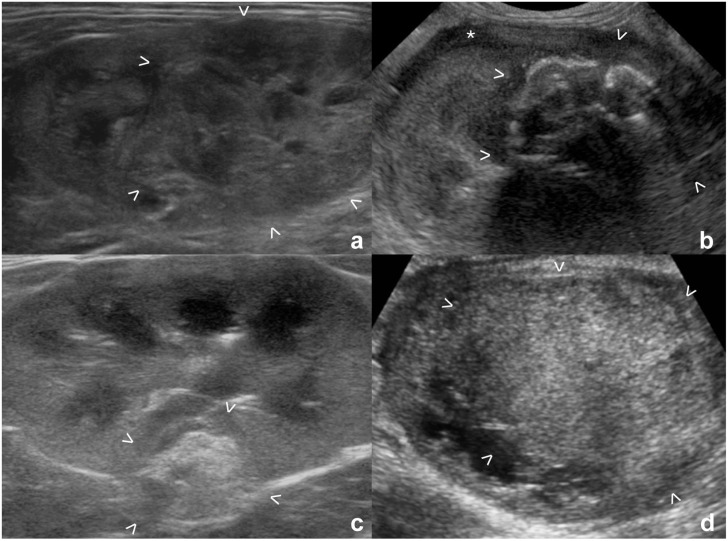
Ultrasonographic appearance of renal masses in four cats: (a,b) carcinoma; (c) adenoma; and (d) nephroblastoma. (a) Heterogeneously hyperechoic renal mass at the caudal pole, with multiple cystic/cavitated areas; (b) heterogeneous mass with markedly hyperechoic shadowing interface (mineralization) and hypoechoic subcapsular rim (*); (c) small hyperechoic mass in the mid portion of the cortex; and (d) mildly heterogeneously hyperechoic mass at the caudal pole. All masses are between arrowheads

A single mass was present in most of the kidneys with carcinoma (42/59, 71%), the three kidneys with adenoma and the single kidney with nephroblastoma (Figure 1). The frequencies of US characteristics of the kidneys with a solitary renal mass in the different tumor types are summarized in Table 2.

**Table 2:** Ultrasonographic characteristics of kidneys with a single renal mass (the total number corresponds to the number of kidneys)*

	Lymphoma (n = 42)	Carcinoma (n = 42)	Sarcoma (n = 7)	Adenoma (n = 3)	Nephroblastoma (n = 1)	*P* value
Echogenicity						
Hypoechoic	36	17	6	1	0	**0.001**
Hyperechoic	3	7	1	2	1	
Isoechoic	3	13	0	0	0	
Mixed	0	5	0	0	0	
Heterogeneous echotexture	13	31	6	2	1	**<0.001**
Mineralization	0	4	1	0	0	0.17
Cavitation	2	7	2	0	0	0.29
Localization						
Whole kidney	1	5	2	0	0	**0.02**
Subcapsular	4	4	1	0	0	
Pelvis/around pelvis	9	2	0	0	0	
Cranial pole	17	15	2	0	0	
Caudal pole	7	16	2	2	1	
Mid portion	4	0	0	1	0	
Distension of the renal pelvis						
Mild	14	2	0	0	0	**0.001**
Moderate	3	10	3	0	1	
Severe	0	4	0	0	0	
Hypoechoic subcapsular rim	26	12	2	0	0	**0.004**

Significant findings are in bold

* Lymphoma was characterized by hypoechoic masses and subcapsular rim, carcinoma by heterogeneity of the mass; distension of the renal pelvis was mainly mild in cases of lymphoma and moderate or severe in carcinoma. None of the histiocytic sarcomas included presented with a single renal mass

**Figure 2:**
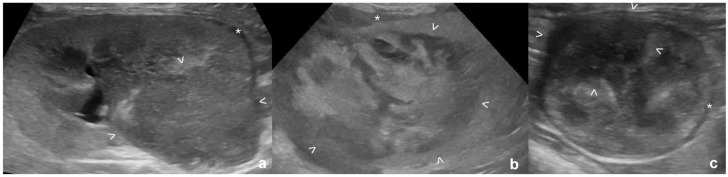
Presence of subcapsular rim in cats with renal masses: (a) lymphoma; (b) carcinoma; and (c) sarcoma. (a) Hypoechoic mass at the caudal pole, thin subcapsular rim (*); (b) markedly heterogeneous mass with mixed echogenicity at the cranial pole, thick and irregular subcapsular rim (*); and (c) mildly heterogeneous markedly hypoechoic mass at the cranial pole, thin circumferential subcapsular rim (*). All masses are between arrowheads

**Figure 3:**
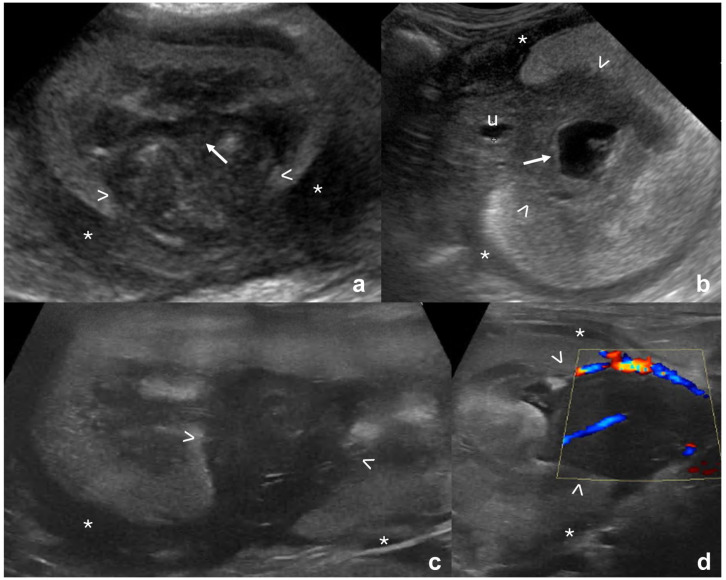
Hypoechoic masses at the renal hilum, continuous with the hypoechoic subcapsular rim, in four cats with renal lymphoma. Thick mass-like hypoechoic tissue (between arrowheads in all images) confluent with hypoechoic subcapsular rim (*) which is thick in panels (a,c) and thinner in panels (b,d). The masses surround the (a) mildly and (b) moderately distended renal pelvis (arrows) and (d) the renal vasculature

A concurrent hypoechoic subcapsular rim was seen in addition to the mass in several cases with lymphoma, carcinoma and sarcoma (Table 2, Figure 2), but was significantly more frequent with lymphoma (*P* = 0.004). Masses were more commonly hypoechoic with lymphoma and sarcoma, while echogenicity was more variable with other tumor types (*P* = 0.001). Echotexture was more commonly homogeneous in lymphoma cases compared with other tumor types (*P* <0.001). Renal length and maximum size of the mass were not different between tumor types (*P* = 0.5 and *P* = 0.48, respectively). There was a significant difference in lesion location across the tumor types, with most carcinomas located at the renal poles (*P* = 0.02), while location was more variable in other tumor types. In 9/42 (21%) cases of lymphoma in which a single mass was present, the mass was located at/around the renal pelvis, with only two (4%) carcinomas and none of the other tumor types detected at this location. In all the lymphoma cases with a mass in this region, the mass was continuous with the hypoechoic subcapsular rim (Figure 3). Pyelectasia was more pronounced in cases of carcinoma or sarcoma compared with lymphoma (*P* = 0.001).

A binomial logistic regression analysis was performed to identify predictors of lymphoma diagnosis vs carcinoma when a renal mass was present. Among all variables, heterogeneity of the mass was the only significant predictor, with heterogeneous echotexture having 4.5 times higher odds of carcinoma (odds ratio [OR] 4.55, 95% confidence interval [CI] 1.75–11.80; *P* = 0.002).

#### US features when a single renal mass was not present (diffuse, multifocal changes)

**Figure 4:**
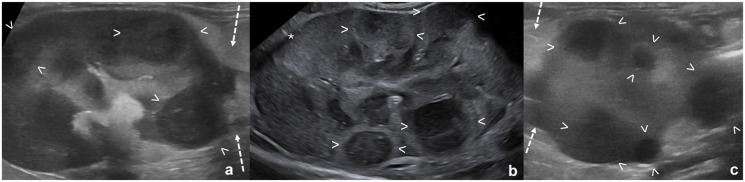
Multiple nodules in cats with different tumors: (a) lymphoma; (b) sarcoma; and (c) histiocytic sarcoma. (a,c) The nodules are homogeneously hypoechoic; and (b) the nodules are heterogeneously hypoechoic. All nodules are between arrowheads. (a,c) Retroperitoneal effusion and steatitis are present (dotted arrows)

**Figure 5:**
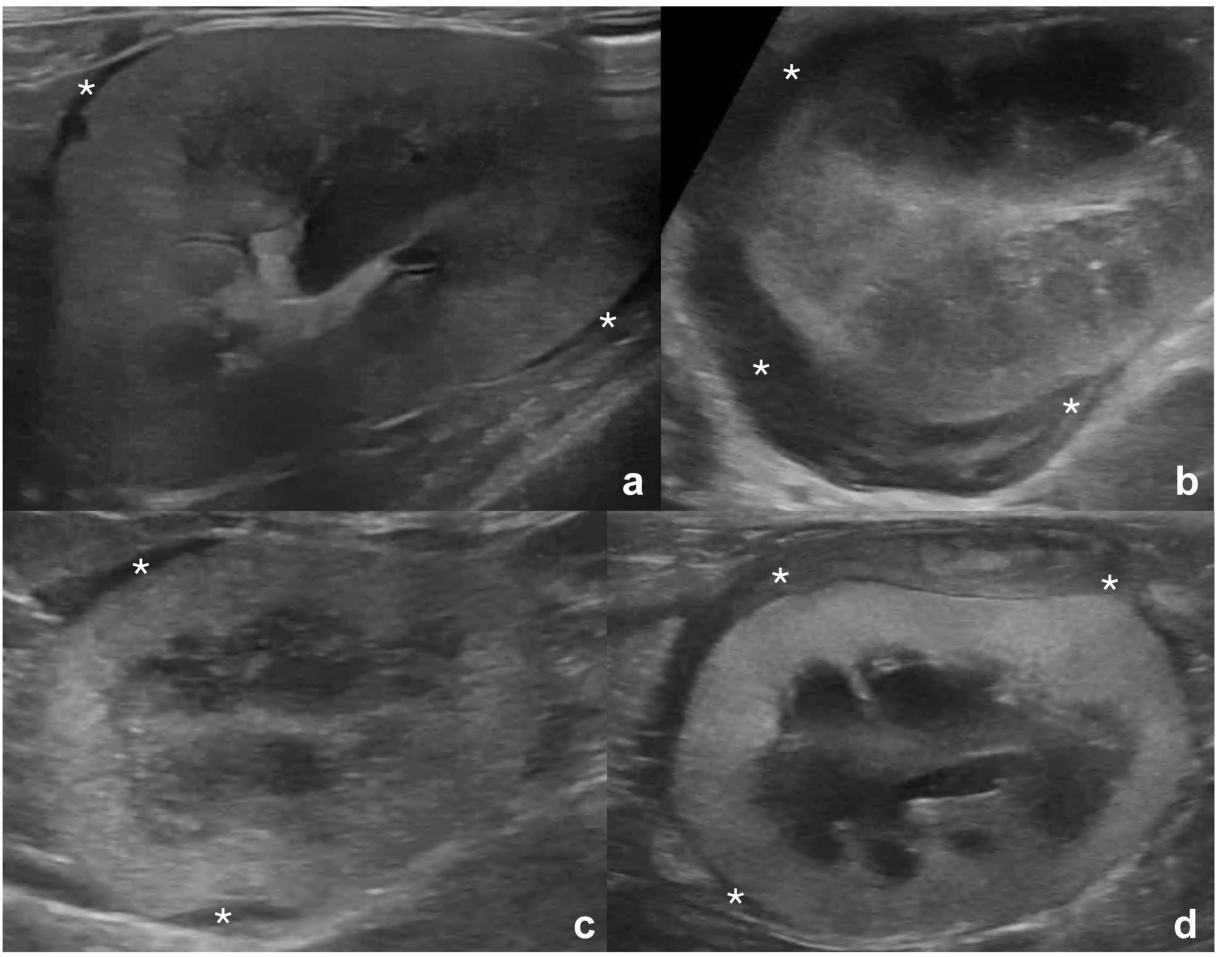
Hypoechoic subcapsular rim in kidneys with diffuse changes in four different cats: (a,b) lymphoma; and (c,d) carcinoma. (a) Thin hypoechoic subcapsular rim (*), with small indentation towards the renal cortex; (b) thick, asymmetric, heterogeneously hypoechoic subcapsular rim (*) at the cranial pole; (c) thin, hypoechoic subcapsular rim (*); and (d) thick, asymmetric hypoechoic subcapsular rim, heterogeneous

The US characteristics of kidneys where a single mass was not present are summarized in Table 3. In kidneys without a renal mass, nephromegaly was more common in cases of lymphoma; in addition, kidneys measured significantly longer in lymphoma cases (*P* = 0.02). Multiple nodules were more common in sarcoma cases (*P* <0.001) (Figure 4). A hypoechoic subcapsular rim was more commonly seen with lymphoma or carcinoma (*P* = 0.01) (Figure 5). Pyelectasia was more commonly mild in lymphoma cases, while moderate/severe pyelectasia was more commonly seen with carcinomas (*P* = 0.002).

**Table 3:** Ultrasonographic characteristics of kidneys where a single renal mass was not present (the total number corresponds to the number of kidneys)*

	Lymphoma (n = 165)	Carcinoma (n = 17)	Sarcoma (n = 7)	Histiocytic sarcoma (n = 3)	*P* value
Nephromegaly	130	8	2	2	**0.001**
Renal length (mm)	51.3 ± 9.8	46.1 ± 6.4	42.8 ± 8.9	48 ± 8.5	**0.02**
Multiple nodules	59	2	6	2	**<0.001**
Hypoechoic subcapsular rim	129	14	3	1	**0.01**
Distension of the renal pelvis					
Mild	72	7	2	2	**0.002**
Moderate	6	3	0	1	
Severe	0	2	0	0	

Data are n or mean ± SD. Significant findings are in bold. None of the adenomas or nephroblastoma included presented without a renal mass

* Cases with lymphoma were more frequently characterized by nephromegaly and hypoechoic subcapsular rim. Multiple nodules were more frequent in cases of sarcomas, whereas renal distension was more severe in cases with carcinoma

A binomial logistic regression was performed to identify predictors of diagnosis of lymphoma vs carcinoma when a renal mass was not present. The presence of nephromegaly (OR 0.09, 95% CI 0.02–0.40; *P* = 0.002) and nodules (OR 0.07, 95% CI 0.01–0.82; *P* = 0.034) were both associated with lower odds of carcinoma, while pelvic dilation was predictive of carcinoma (OR 3.73, 95% CI 1.39–10.0; *P* = 0.009).

Retroperitoneal effusion and steatitis were commonly detected with all diagnoses with no statistical difference between tumor groups.

### Bilateral renal changes and additional organ involvement

Details on additional organ changes in different tumor types are reported in Table 4. Most of the cases (175/187, 93.5%) underwent complete abdominal US; in 105/187 (56%) cases, thoracic imaging results were also available and 12 additional cases had necropsy performed.

**Table 4:** Frequency of involvement of additional organs and changes in renal parameters among tumor types in the cats included in the study*

		Lymphoma (n = 118)	Carcinoma (n = 53)	Sarcoma (n = 10)	Histiocytic sarcoma (n = 2)	Adenoma (n = 3)	Nephro blastoma (n = 1)	*P* value (lymphoma vs carcinoma)
Additional organs affected								
Total^†^		72/118	19/53	6/10	1/2	0	0	**0.026**
GI tract		35	–	–	–	–	–	
Liver/spleen		15	3	2	1	–	–	
Lymph nodes		12	–	1	1	–	–	
Peritoneum/retroperitoneum		2	9	–	–	–	–	
Pulmonary nodules		–	2	1	–	–	–	
Thrombosis		–	3	–	–	–	–	
Other sites		Nose (4), mediastinum (1), pharynx (1), eye (1)	Bladder (1)	Mediastinum (1), thoracic wall (1)	–	–	–	
Clinical chemistry and urinalysis								
Elevated creatinine		64/106	11/46	1/9	1/2	1/3	–	**<0.001**
Elevated BUN		66/106	12/46	2/9	2/2	1/3	–	**<0.001**
USG <1.035		54/68	21/34	2/5	1/1	0/1	–	0.057

Serum creatinine and blood urea nitrogen (BUN) concentrations exceeding the reference intervals provided by the respective laboratories were classified as elevated. Significant results are in bold

* In some cats, more than one organ/apparatus was concurrently affected. Lymphoma showed frequently concurrent involvement of additional organs and elevated levels of serum creatinine and BUN

† The total refers to the number of cats

GI = gastrointestinal; USG = urine specific gravity

Changes in additional organs were detected in 98 cases and were more common with lymphoma (*P* = 0.026): 72/118 (61%) cats with lymphoma presented lesions in multiple organs, indicating a multicentric form of the disease. Of these, the majority had a concurrent gastrointestinal mass. In nine cats with renal carcinoma, peritoneal and/or retroperitoneal nodules (carcinomatosis) were present. Three cats with carcinoma had thrombosis in the aorta, caudal vena cava and renal vein (one case each).

### Clinicopathological data

Serum chemistry profiles were available for 166 cases and urinalysis was available for 109 cases (Table 4). Elevation in serum creatinine and BUN concentrations, along with USG below 1.035, were more frequently observed in lymphoma compared with carcinoma. Of the 78 cats with USG below 1.035, four were hyposthenuric (USG <1.008) and all were diagnosed with lymphoma.

The results of the logistic regression performed on the lymphoma group to assess the effect of age, bilateral renal involvement and type of lymphoma on the odds of having elevated serum creatinine and BUN concentrations, as well as USG below 1.035 are shown in Table 5. Bilateral lymphoma was associated with increased odds of elevated serum BUN concentration (*P* = 0.04). These patients also tended to show higher odds of elevated serum creatinine and USG below 1.035; however, these differences were not statistically significant. Conversely, although not statistically significant, cats with focal/multifocal renal lymphoma tended to show lower odds of elevated serum creatinine, BUN and USG below 1.035 compared with those with diffuse lymphoma.

**Table 5:** Logistic regression model results showing the effect of age, bilateral disease and focal type of lymphoma (single mass or nodules) on changes in serum creatinine and blood urea nitrogen (BUN) concentrations, as well as urine specific gravity (USG)

	Elevated creatinine		Elevated BUN		USG <1.035	
	OR (95% CI)	*P* value	OR (95% CI)	*P* value	OR (95% CI)	*P* value
Age	1.01 (0.9–1.1)	0.83	0.99 (0.8–1.1)	0.98	1.14 (0.9–1.3)	0.12
Bilateral disease	1.83 (0.7–4.6)	0.21	2.5 (1–6.4)	**0.04**	1.97 (0.5–7.3)	0.3
Focal/multifocal type of lymphoma	0.59 (0.2–13.9)	0.19	0.58 (0.2–1.3)	0.53	0.67 (0.1–2.5)	0.93

Significant results are in bold. Bilateral renal lymphoma is associated with increased odds of elevated BUN

CI = confidence interval

## Discussion

This study described the US characteristics of renal neoplasia in a large group of cats. The most common tumor was lymphoma, followed by carcinoma.

The most common features in feline renal lymphoma were nephromegaly and hypoechoic subcapsular rim, both of which were present in more than three-quarters of the included cats with this diagnosis. In most of the lymphoma cases, the changes were bilateral. Renal enlargement was previously reported as a common feature in cats with renal lymphoma.^19–21,24^ In the present study, nephromegaly occurred in cases of lymphoma, both with and without a renal mass. Interestingly, when a single renal mass was not present, the size of the kidney was significantly larger in cases with lymphoma than in cases with carcinoma or sarcoma. The nephromegaly observed in lymphoma likely reflects diffuse lymphocytic infiltration within the renal parenchyma,^27^ which may be reversible with appropriate chemotherapy.^28^

The hypoechoic subcapsular rim was also present in 75% of the kidneys with confirmed renal lymphoma. As previously reported, this hypoechoic tissue likely represents subcapsular lymphomatous infiltrate.^21^ In some of the lymphoma cases, a hypoechoic mass, continuous with the hypoechoic subcapsular rim and with the same echogenicity, was detected at the level of the renal hilum, surrounding the renal pelvis. This specific location follows the path of the renal lymphatics described in humans, where the lymph, from the cortical intralobular, arcuate and interlobar lymphatics, can direct both toward the hilum and toward the capsular lymphatic plexus, the two routes that lymph uses to drain out of the kidney.^29,30^ The hilar lymphatic vessels are located adjacent to the renal arteries and veins,^29,30^ where the masses/hypoechoic thickening were visible in our study. This location, together with the echogenicity of the tissue (hypoechoic, similar to the hypoechoic subcapsular rim), should increase the suspicion of renal lymphoma in cats.

Although less common, a hypoechoic subcapsular rim was also observed in carcinoma and sarcoma cases, both with and without masses, and was detected in one case of histiocytic sarcoma. Recently, subcapsular thickening has been described in feline patients with metastatic carcinoma in the kidneys, and histology in some of those cases revealed necrotic tissue as well as neoplastic cells.^23^ Therefore, while the hypoechoic subcapsular rim is significantly more frequent in cases of lymphoma, it is not pathognomonic for this disease and should be interpreted together with other US findings. Given its relatively superficial location and potential for containing neoplastic cells,^21,23^ this region is particularly suitable for US-guided sampling to obtain a definitive diagnosis.

Consistent with previous reports, the presence of a single renal mass was the most common presentation in cats with renal carcinoma.^17,31^ The few cases of renal adenoma also presented as a single mass, as described in previous reports.^4,6^ Although detected in other tumor types, the presence of a single mass appears, therefore, more frequently associated with an epithelial tumor. Furthermore, logistic regression analysis showed that a heterogeneous echotexture of the renal mass was associated with a 4.5-fold higher likelihood of carcinoma compared with lymphoma. Interestingly, lymphoma presented as a single mass in 20% of the cases; therefore, this sonographic feature should not lead to the exclusion of lymphoma as a differential in the clinical setting when a renal mass is present, particularly if hypoechoic and in a particular location (around the renal pelvis).

In contrast, multiple nodules were more common in sarcoma, histiocytic sarcoma and lymphoma. Renal sarcomas are rare in cats and, according to previous reports, typically appear as unilateral or bilateral masses.^7–9^ In the current study, about half of the confirmed sarcomas presented as single hypoechoic, heterogeneous masses and the remainder as multiple nodules. Because necropsy confirmation was not available in all cases with lesions in other organs, some could potentially represent metastatic disease rather than primary renal tumor, which may explain the higher frequency of nodular appearance observed. A nodular appearance is the most common feature of renal lymphoma in humans^32^ and has been reported in cats.^20,28,31,33^ Nevertheless, the frequency of renal nodules may be higher, given the possibility of underestimating the presence and number of renal nodules with US.^33^ The appearance of the included histiocytic sarcomas, nodular and with hypoechoic subcapsular rim, was similar to an earlier case.^11^

Pyelectasia was a common finding across all tumor types, both in kidneys with and without renal masses. Overall, cats with carcinoma or sarcoma tended to have a greater degree of pelvic dilation compared with lymphoma cases. The dilation of the renal pelvis in our population of cats could be mainly explained with either a partial obstruction secondary to a compression of a renal mass on the collecting system and/or with an acute kidney injury secondary to the neoplasia.^34^ However, mild pelvic distension can be observed in cats with normal renal function, under normal conditions and/or when receiving intravenous (IV) fluids.^35^ Because of the retrospective nature of the study, information regarding previous IV fluid administration was not available.

In addition, despite the more severe pelvic dilation observed in the carcinoma group, serum creatinine and BUN concentrations were significantly more frequently high in the lymphoma group, suggesting a greater impairment of renal function in cats with renal lymphoma compared with those with carcinoma. This difference is likely explained by the more common bilateral renal involvement in cats with renal lymphoma compared with carcinoma (75% vs 11%) and potentially also by the more diffusely infiltrative nature of lymphoma. USG below 1.035 was observed in a larger number of patients with renal lymphoma than with carcinoma, although this difference was less marked and not statistically significant. Multiple factors other than renal disease have been detected to influence USG in cats, including age and diet, and some healthy cats can present with USG below 1.035 without overt pathology.^25^

When considering only the lymphoma group, 60% of cats were azotemic and bilateral renal neoplasia increased the odds of elevated serum BUN concentrations by 2.5-fold, with a similar (although not statistically significant) trend observed for serum creatinine. This further supports the hypothesis of bilateral disease playing a major role in the higher frequency of renal function impairment in cats with lymphoma compared with carcinoma (only 24% of cats were azotemic). Within the lymphoma group, age was not associated with elevations in serum creatinine or BUN concentrations, and no significant differences were found between the different tumor groups. The sonographic appearance of the lymphoma, focal/multifocal vs diffuse, did not show a statistically significant difference, although focal forms generally appeared to have lower odds of renal function impairment.

This study has some limitations. Its retrospective design relied on existing medical records and imaging archives, which introduces variability in data quality and completeness. Data were collected from several universities over a 14-year period involving various US machines and operators, which could prevent the detection of some US features. Many diagnoses were based on cytology, with histopathological confirmation not available for all cases; this may limit diagnostic certainty. Not all cats in our study had complete clinicopathologic panels and full staging available; furthermore, clinicopathological data were compared individually and not assessed in combination, which differed from the evaluations that could be made in a clinical context. In addition, information on prior treatments, such as IV fluids, which could affect both renal US appearance and function, was not consistently available. Tumors like nephroblastoma, adenoma and histiocytic sarcoma were very uncommon in our population, which prevents us from drawing conclusions regarding their US features, restricts the generalizability of our findings for these tumor types and limits our statistical comparisons. Another limitation is that kidneys from the same cat were analyzed as separate observational units to compare US features. Although this approach reflects the organ-specific nature of imaging findings, it may introduce within-patient clustering effects, particularly in cats with bilateral disease. This potential non-independence should be considered when interpreting the statistical results, especially for tumor types with a high frequency of bilateral involvement, such as lymphoma. Further prospective standardized studies with comprehensive diagnostic confirmation and follow-up are recommended to validate our findings.

It is important to note that the US findings described are not specific to neoplastic disease. Nephromegaly, hypoechoic subcapsular rim and renal nodules have also been reported in cats with feline infectious peritonitis,^21,36^ and renal masses identified on US may also represent benign lesions, such as hematomas or abscesses.^20,37^ Further studies are therefore warranted to evaluate the diagnostic performance of abdominal ultrasonography in differentiating neoplastic from non-neoplastic renal conditions in cats.

## Conclusions

Lymphoma was the most common renal tumor in cats; nephromegaly and a hypoechoic subcapsular rim were the most common US features of renal lymphoma, reflecting diffuse lymphocytic infiltration. Lymphoma was commonly associated with bilateral disease and higher frequency of azotemia on presentation. Carcinomas most often presented as single, heterogeneous renal masses and with more marked renal pyelectasia. Multiple nodules were observed in sarcoma and lymphoma, and half of the sarcomas presented as a single, hypoechoic, heterogeneous mass.

Overall, the combination of US findings, including bilateral involvement, presence of single mass vs multiple nodules, hypoechoic subcapsular rim and nephromegaly, may provide valuable guidance in distinguishing feline renal tumor types.
